# Simplified Interval Observer Scheme: A New Approach for Fault Diagnosis in Instruments

**DOI:** 10.3390/s110100612

**Published:** 2011-01-10

**Authors:** Albino Martínez-Sibaja, Carlos M. Astorga-Zaragoza, Alejandro Alvarado-Lassman, Rubén Posada-Gómez, Gerardo Aguila-Rodríguez, José P. Rodríguez-Jarquin, Manuel Adam-Medina

**Affiliations:** 1 Instituto Tecnológico de Orizaba, División de Estudios de Posgrado e Investigación, Av. Oriente 9 No. 852, Col. Emiliano Zapata, 94320 Orizaba, Veracruz, Mexico; E-Mails: lassman@prodigy.net.mx (A.A.-L.); rposada@itorizaba.edu.mx (R.P.-G.); gerardo_aguila03@yahoo.com.mx (G.A.-R.); jprj_02@hotmail.com (J.P.R.-J.); 2 Centro Nacional de Investigación y Desarrollo Tecnológico, Departamento de Ingeniería Electrónica, Interior Internado de Palmira s/n, 69490 Cuernavaca, Morelos, Mexico; E-Mails: astorga@cenidet.edu.mx (C.M.A.-Z.); adam@cenidet.edu.mx(M.A.-M.)

**Keywords:** ADM1, interval observer, UASB reactor

## Abstract

There are different schemes based on observers to detect and isolate faults in dynamic processes. In the case of fault diagnosis in instruments (FDI) there are different diagnosis schemes based on the number of observers: the Simplified Observer Scheme (SOS) only requires one observer, uses all the inputs and only one output, detecting faults in one detector; the Dedicated Observer Scheme (DOS), which again uses all the inputs and just one output, but this time there is a bank of observers capable of locating multiple faults in sensors, and the Generalized Observer Scheme (GOS) which involves a reduced bank of observers, where each observer uses all the inputs and m-1 outputs, and allows the localization of unique faults. This work proposes a new scheme named Simplified Interval Observer SIOS-FDI, which does not requires the measurement of any input and just with just one output allows the detection of unique faults in sensors and because it does not require any input, it simplifies in an important way the diagnosis of faults in processes in which it is difficult to measure all the inputs, as in the case of biologic reactors.

## 1. Introduction

Processes supervision systems for operators have evolved as new techniques of detection and isolation of faults have appeared. Research in this field has also grown as the complexity of industrial processes has grown and this has motivated the development of different focuses for FDI system design.

The diagnosis of faults can be done using observers. One great advantage of the diagnosis schemes based on observers is that in comparison with other methods they are very large schemes. The high level of complexity in current industrial processes has led to a situation where the amount of information generated by these processes can overcome the capacity of analysis of human operators, which hinders decision making [1,2]. The most recent supervision systems have the capacity to carry out diagnosis and maintenance functions, in order to guarantee the correct functionality of highly complex processes [3–8].

As an example of a process that is hard to supervise, in this work the production of biogas in an anaerobic reactor is used as a case study in which faults are diagnosed and isolated using a scheme based on observers. In many publications about non linear observers for the design of FDI systems, the residuals are based in the error of the estimation obtained by the observer [9]. In biological processes, due to their non linear nature, in the majority of cases they are not completely observable, therefore it is more appropriate to consider some relationships among parameters, instead of attempting to estimate them individually [10,11]. The work presented in [11] explores a methodology to determine the global state and parameters of biological reactors. The method proposed in the article allows one to formalize the design of asymptotic observers, which are capable of evaluating certain variables of state, which are not measured for the anaerobic digestion process, despite certain doubts about the kinetics of the process. We should mention that asymptotic observers need the information of all the input variables of the process, which it is practically impossible to achieve under normal conditions in the operation of an anaerobic reactor. Overcoming this problem implies knowing all the input variables of an anaerobic reactor and for this purpose an observer by intervals was developed in [12]. The main characteristic of intervals observers is that they are capable of providing estimations of guaranteed intervals of non measured state variables instead of an exact estimation, if a superior and inferior limit is provided for each one of the input processes.

## 2. Fault Diagnosis Schemes

Diagnosis schemes based on observers can be classified according the type of fault detected: sensor faults (Instrument Fault Detection or IFD), actuator faults (Actuator Fault Detection or AFD), and component faults (Component Fault Detection or CFD). Diagnosis schemes can also be classified according the number of observers that are used. There are schemes with one observer: a *Direct scheme* is a scheme of just one observer of complete order. The *Simplified Observer Scheme* (SOS), is a scheme of one observer of reduced order. For sensor faults (IFD), the only observer in this scheme uses all the inputs and one output, which only provides simple redundancy and only allows the localization of faults in one sensor. In AFD, the only observer uses all the outputs and just one input. When several observers constitute a bank of observers of reduced order we have a *Dedicated Observer Scheme* (DOS). For faults in sensors (IFD), each observer uses all the inputs and just one output. The number of observers equals the number of outputs (sensors). For actuator faults (AFD) each observer uses one input and all the outputs. It should be mentioned that the DOS scheme allows the localization of multiple faults, either in sensors (IFD) or in actuators (AFD). The *Generalized Observer Scheme* (GOS) is formed by a bank of observers of reduced order. For faults in sensors (IFD), each observer uses all the inputs and m-1 outputs, where m is the number of outputs. For actuator faults (AFD), each observer uses all the outputs and n-1 inputs, n being the number of inputs.

## 3. Design and Implementation of a New Diagnosis Scheme: SIOS-IFD

The SIOS-IFD is a scheme with just one interval observator, of reduced order, for faults in sensors. The main advantage of the SIOS-IFD scheme over all the previously presented schemes, is the fact that no input measurements are is required; it is only necessary to have knowledge of the interval of values that the named inputs can reach. SIOS-IFD only allows the localization of faults in one sensor, because it requires the in line measurement of just one output.

**Figure 1.**
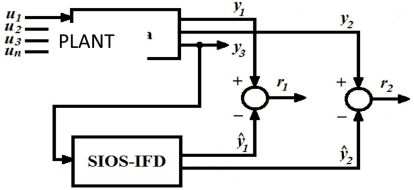
SIOS-IFD.

Figure 1 shows a block diagram of the SIOS-IFD. In this figure it can be observed that the SIOS-IFD does not use the inputs *u_i_* *(I = 1,2,3,4,…,n)*, but rather just uses one output *(y_3_)* to estimate the other two outputs (*ŷ*_1_ e *ŷ*_2_) and in that way be able to generate the responses: *r*_1_ = *y*_1_ – *ŷ*_1_ y *r*_2_ = *y*_2_ – *ŷ*_1_.

## 4. Mathematical Model of the Anaerobic Reactor Used as Case Study

**Figure 2.**
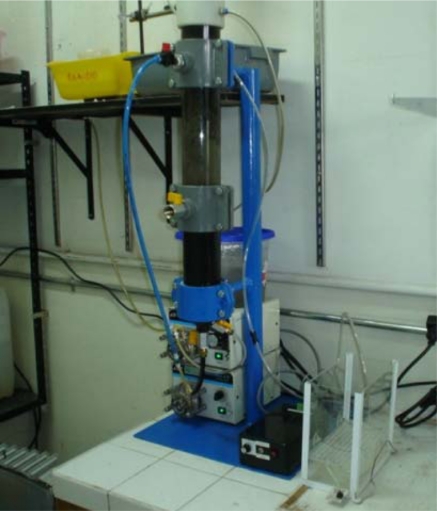
UASB reactor.

Next the simplified ADM1 mathematic model of the UASB reactor of the Instituto Tecnológico de Orizaba, Veracruz, México (Figure 2), which is the case study of the project of this article, is presented.

Simplified ADM1 Model:

(1) $$\begin{array}{c} {\dot{x}}_{1}=Y_{1}K_{m,1}\frac{s_{1}}{K_{s,1}+s_{1}}I_{\mathrm{pH}}\;x_{1}-\mathrm{aD}(t)x_{1}-K_{d}\;x_{1}\\ {\dot{s}}_{1}=D(s_{1}^{i}-s_{1})-K_{m1}\frac{s_{1}}{K_{s,1}+s_{1}}I_{\mathrm{pH}}\;x_{1}\\ {\dot{Q}}_{\text{CH}4}=(1-Y_{1})Y_{\text{CH}4}\;K_{m1}\frac{s_{1}}{K_{s,1}+s_{1}}I_{\text{pH}}x_{1}-Q_{\text{CH}4} \end{array}$$

with:

$$\begin{array}{l} \mu_{1}=\frac{K_{m,1}s_{1}}{K_{s,1}+s_{1}}I_{\mathrm{pH}}\\ I_{\mathrm{pH}}=\frac{1+2*10^{0.5({\mathrm{pH}}_{\mathrm{LL}}-{\mathrm{pH}}_{\mathrm{UL}})}}{1+10^{\left(\mathrm{pH}-{\mathrm{pH}}_{\mathrm{UL}}\right)}+10^{\left({\mathrm{pH}}_{\mathrm{LL}}-\mathrm{pH}\right)}} \end{array}$$

where *x*_1_ is the concentration of the anaerobic mass, *s*_1_ is the concentration of organic matter expressed as chemic oxygen demand (COD), *Q_CH4_* is the exiting flux of methane biogas, *D(t)* is the rate of dilution, *K_m1_*, *K_d_* y *K_s1_* are the specific rates of growth of anaerobic mass, the dilution rate of the anaerobic reactor and the constant decrease of semisaturation for the anaerobic biomass, respectively. *Y_1_* is the coefficient of performance for the degradation of COD, $s_{1}^{i}$ is the concentration of COD in the affluent, *I_PH_* represents the *pH* inhibition, where *pH_LL_* and *pH_UL_* are the lower and higher *pH* limits, respectively. The values of the model parameters are shown in Table 1.

## 5. Interval Observer Designed for the SIOS-IFD Scheme

In this section the design of the interval observer designed for the IOS – IFD scheme is presented. The designed interval observer is capable of stimating value *x_1_* y *Q_CH4_* from the in-line measurement of *s_1_*. It should be emphasized that the designed interval observer does not require any measurement of the reactor input variables. The first necessary condition for the design of an interval observer is that a hypothetic observer of known inputs must exist, called base observer. In order to satisfy this first condition, the asymptotic observer presented next was designed.

The model described by the set of differential non linear Equation (1) can be rewritten in the following way:

(2) $$\dot{x}(t)={\left[\begin{array}{lll} {\dot{x}}_{1} & {\dot{Q}}_{\mathrm{CH}4} & {\dot{s}}_{1} \end{array}\right]}^{T}=\mathrm{Cf}(x(t),\;t)+A(t)x(t)+b(t)$$

with:

$$\begin{array}{l} f(x(t),\;t)=[\mu_{1}x_{1}]\\ C={\left[\begin{array}{ccc} C_{1}^{T} & \vdots & C_{2}^{T} \end{array}\right]}^{T}={\left[\begin{array}{llll} Y_{1} & (1-Y_{1})Y_{\mathrm{CH}4} & \vdots & -1 \end{array}\right]}^{T}\\ A(t)=\left[\begin{array}{ccc} A_{11}(t) & \vdots & A_{12}(t)\\ \cdots & \vdots & \cdots \\ A_{21}(t) & \vdots & A_{22}(t) \end{array}\right]=\left[\begin{array}{cccc} -\left(\mathrm{aD}(t)+k_{d}\right) & 0 & \vdots & 0\\ 0 & -1 & \vdots & 0\\ \cdots & \cdots & \cdots & \cdots \\ 0 & 0 & \vdots & -D(t) \end{array}\right]\\ b(t)=[\begin{array}{lll} b_{1}^{T}(t) & \vdots & b_{2}^{T}(t) \end{array}]=D(t){\left[\begin{array}{lll} 0 & 0 & \vdots s_{1}^{i}(t) \end{array}\right]}^{T} \end{array}$$

where *x*(*t*) ∈ ℜ*^n^* is the state vector, C ∈ ℜ*^m×n^* is the matrix of performance coefficients and f(*x*(*t*), t) ∈ ℜ*^m^* is the vector that contains the non linearity of the model, which are assumed to be totally unknown, the time variant matrix *A*(*t*) ∈ ℜ*^n×n^* is the matrix of state and *b*(*t*) ∈ ℜ*^n^* is the vector of the observer entries.

The asymptotic observer is designed under the assumption that all inputs are known, and *m* measures states on-line. Thereby the the space of states can be divided in such way that Equation (2) can be rewritten as:

$$\begin{array}{l} {□}_{1}(t)=C_{1}f(x(t),t)+A_{11}(t)v_{1}(t)+A_{12}(t)v_{2}(t)+b_{1}(t)\\ {□}_{2}(t)=C_{2}f(x(t),t)+A_{21}(t)v_{1}(t)+A_{22}(t)v_{2}(t)+b_{2}(t) \end{array}$$

where the *m* measured states are regrouped in vector *v_2_(t) (dim v_2_(t) = m)* and the variables that will be estimated are represented by *v_1_(t) (dim v_1_(t) = s = n – m)*. The matrices *A*_11_(*t*) ∈ ℜ^*s*×*s*^, *A*_12_(*t*) ∈ ℜ^*s*×*m*^, *A*_21_(*t*) ∈ ℜ^*m*×*s*^, *A*_22_(*t*) ∈ ℜ^*m*×*m*^, *C*_1_ ∈ ℜ^*s*×*r*^, *C*_2_ ∈ ℜ^*m*×*r*^, *b*_1_ ∈ ℜ*^s^* *y b*_2_ ∈ ℜ*^m^* are the corresponding partitions of *A*(*t*), *C y b*(*t*), respectively.

Equation (3) represents the asymptonic observer that was designed for the anaerobic reactor described for the Equation (1):

(3) $$\begin{array}{c} \dot{\hat{w}}=W(t)\hat{w}(t)+𝒼(t)v_{2}(t)+\mathrm{Nb}(t)\\ \hat{w}(0)=N_{1}{\hat{v}}_{1}(0)+N_{2}{\hat{v}}_{2}(0)\\ {\hat{x}}_{1}(t)=N_{1}^{-1}(\hat{w}(t)-N_{2}v_{2}(t))\\ \left[\begin{array}{c} {\hat{y}}_{1}(t)\\ {\hat{y}}_{2}(t) \end{array}\right]=\left[\begin{array}{cc} 1 & 0\\ 0 & 1 \end{array}\right]{\hat{x}}_{1}(t) \end{array}$$

with:

$$\begin{array}{l} W(t)=\left(N_{1}A_{11}(t)+N_{2}A_{21}(t)\right)N_{1}^{-1}\\ 𝒼(t)=N_{1}A_{12}(t)+N_{2}A_{22}(t)-W(t)N_{2} \end{array}$$

In the design of this asymptonic observer, it was assumed that the rate of dilution D, and the inputs to the digestor are considered as knowntherefore A(t) y b(t) are known ∀ *t* ≥ 0. The asymptonic observer 1 was designed assuming *s_1_* as the only state measured on-line, meaning, *v_2_(t) =* [*s_1_*] *(dim v_2_(t) = 1),* while *x_1_* y *s_1_* were assumed as estimated states, meaning *v_1_(t) =* [*x_1_ Q_CH4_*]^T^ *(dim v_1_(t) = 2)*. For the calculation of the matrix *N*, *N_1_* was arbitrarily chosen as an identity matrix, to calculate *N_2_* in the following way: *N*_2_= –*N*_1_*C*_1_*C*_2_^§^, (where *C*_2_^§^ is the pseudo reverse generalization of *C_2_*), thus obtaining the following result:

$$N=[\begin{array}{ccc} N_{1} & \vdots & N_{2} \end{array}]=\left[\begin{array}{cccc} 1 & 0 & \vdots & Y_{1}\\ 0 & 1 & \vdots & (1-Y_{1})Y_{\mathrm{CH}4} \end{array}\right]$$

Substituting *N_1_* and *N_2_* in Equation (3), the following is obtained:

$$W(t)=W_{e}=\left[\begin{array}{cc} -(\mathrm{aD}(t)+k_{d}) & 0\\ 0 & -1 \end{array}\right]$$

Finally, *W_e_^+^* was calculated using the minimum value of the dilution rate *D(t)* = 0.01, obtaining:

$$W_{e}^{+}=\left[\begin{array}{cc} -0.025 & 0\\ 0 & -1 \end{array}\right]$$

*W_e_^−^* was calculated using the maximun value of the dilution value *D(t)* = 1, obtaining:

$$W_{e}^{-}=\left[\begin{array}{cc} -0.52 & 0\\ 0 & -1 \end{array}\right]$$

The eigenvalues of *W_e_^+^* y *W_e_^−^* are, respectively:

$$\begin{array}{l} \lambda (W_{e}^{+})=[\begin{array}{cc} -0.025 & -1 \end{array}]\\ \lambda (W_{e}^{-})=[\begin{array}{cc} -0.52 & -1 \end{array}] \end{array}$$

Now, in order to make the observer asymptotically stable, the following conditions must be fulfilled:

- *W_e_^−^_i,j_* ≥ 0 ∀*i* ≠ *j*.
- *W_e_^+^* *y W_e_^−^* must be stable.

Since both conditions are fulfilled, the observer is asymptotically stable. Next the design of an interval observer based on the designed asymptotic observer is presented. Once the asymptotic observer that will work as base observer have been designed, we continued with the design of the interval observer, which is based in the supposition that the values of the input vector *b(t)* are unknown, but their upper limit *b*^+^*(t)* and lower limit *b*^−^*(t)* are known, so *b*^−^*(t)* ≤ *b(t)* ≤ *b*^+^*(t)*. Thus, the asymptotic observer presented in Equation (3) works as a structure base to build the next interval observer:

For the upper limit:

(4) $$\left\{ \begin{array}{c} {\dot{\hat{w}}}^{+}(t)=W{\hat{w}}^{+}(t)+𝒼(t)v_{2}(t)+M\beta^{+}(t)\\ {\hat{w}}^{+}(0)=N_{1}{\hat{v}}_{1}{}^{+}(0)+N_{2}v_{2}(0)\\ {\hat{x}}_{1}{}^{+}(t)=N_{1}{}^{-1}({\hat{w}}^{+}(t)-N_{2}v_{2}(t))\\ \left[\begin{array}{c} {\hat{y}}_{1}{}^{+}(t)\\ {\hat{y}}_{2}{}^{+}(t) \end{array}\right]=\left[\begin{array}{cc} 1 & 0\\ 0 & 1 \end{array}\right]{\hat{x}}_{1}{}^{+}(t) \end{array}\right.$$

For the lower limit:

(5) $$\{\begin{array}{c} {\dot{\hat{w}}}^{-}(t)=W{\hat{w}}^{-}(t)+𝒼(t)v_{2}(t)+M\beta^{-}(t)\\ {\hat{w}}^{-}(0)=N_{1}{\hat{v}}_{1}{}^{-}(0)+N_{2}v_{2}(0)\\ {\hat{x}}_{1}{}^{-}(t)=N_{1}{}^{-1}({\hat{w}}^{-}(t)-N_{2}v_{2}(t))\\ \left[\begin{array}{c} {\hat{y}}_{1}{}^{-}(t)\\ {\hat{y}}_{2}{}^{-}(t) \end{array}\right]=\left[\begin{array}{cc} 1 & 0\\ 0 & 1 \end{array}\right]{\hat{x}}_{1}{}^{-}(t) \end{array}$$

where:

$$\begin{array}{l} M\;=\;[\begin{array}{lllll} N_{1} & \vdots & N_{2} & \vdots & \left|N_{2,ij}\right| \end{array}]\\ \beta^{+}(t)={\left[\begin{array}{lllll} b_{1}^{+}(t) & \vdots & \frac{1}{2}(b_{2}^{+}(t)+b_{2}^{-}(t)) & \vdots & \frac{1}{2}(b_{2}^{+}(t)-b_{2}^{-}(t)) \end{array}\right]}^{T}\\ \beta^{-}(t)={\left[\begin{array}{lllll} b_{1}^{-}(t) & \vdots & \frac{1}{2}(b_{2}^{+}(t)+b_{2}^{-}(t)) & \vdots & -\frac{1}{2}(b_{2}^{+}(t)-b_{2}^{-}(t)) \end{array}\right]}^{T} \end{array}$$

The convergence of the interval observer is based in the principal of cooperation defined by [13] (see Lema 1). Let *ẽ*^+^(*t*) = *v̂*_1_^+^ – *v*_1_ y *ẽ^−^*(*t*) = *v*_1_ – *v̂*_1_^−^ be the errors of estimation associated with Equations (4) and (5), respectively. The follwoing expression represents the dynamics of those errors:

(6) $$\dot{\tilde{e}}*(t)=W_{e}{\tilde{e}}^{*}(t)+\Omega^{*}$$

It uses ẽ*(*t*) to refer to any of the errors *ẽ^+^*(*t*) o *ẽ^−^*(*t*) so that their dynamics have the same mathematical structure. In the previous equation Ω* = *N*_1_^−1^ *N*(*b*^+^(*t*) – *b*(*t*)) for the case of the upper limit, while Ω* = *N*_1_^−1^ *N*(*b*(*t*) – *b*^−^(*t*)) for the case of lower limit

*Lema 1* [11]

In *ω̇* = *f* (ω, *t*). The system it is cooperative if $\frac{\partial f_{h}(\omega ,t)}{\partial \omega_{d}}\geq \;0\;\;\forall h\;\neq \;d$.

This implies that if *ω*(0) ≥ 0 then *ω*(*t*) ≥ 0 ∀*t* ≥ 0. Therefore, if the initial conditions of the estimated variables are unknown but have their limits organized as: *v̂*_1_^−^(0) ≤ *v*_1_(0) ≤ *v̂*_1_^+^ (0) then ẽ*(0) ≥ 0. Besides, if the system (6) is cooperative, the matrix *W_e_* is stable (Hurwitz) and if Ω* is positive (or zero), it can be guaranteed that ẽ*(*t*) ≥ 0 ∀*t* ≥ 0, and in consequence: *v̂*_1_^−^(*t*) ≤ *v*_1_(*t*) ≤ *v̂*_1_^+^(*t*) ∀*t* ≥ 0 [12].

## 6. Experimental Results of the Interval Observer

**Figure 3.**
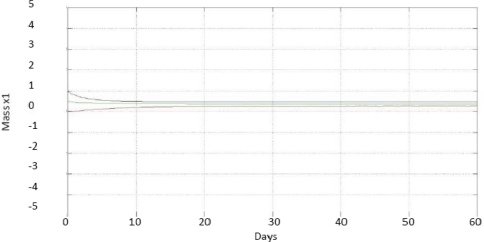
Estimation of the concentration of anaerobic mass x1.

**Figure 4.**
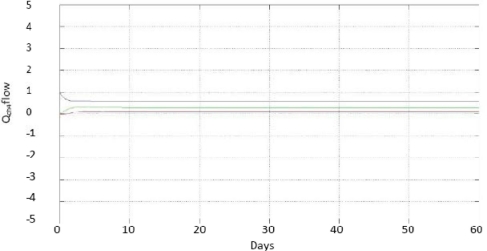
Estimation of methane biogas flow *Q_CH4_*.

To experimentally verify the operation of the developed interval observer, the anaerobic digester was fed with wastewater from a brewery, which had maximum values of 3 gCOD/L and minimum values of 2 g COD/L, these values are based to the digester being accustomed to consume 3 gCOD/L. If the concentration of the water is greater than this, it can be diluted to achieve the desired value, but if it is lesser, it will be difficult to remove excess water to achieve the desired value, being in the worst case a concentration of 2 gCOD/L. The dilution rate is bounded by a maximum value of 0.74 d^−1^ and minimum value of 0.26 d^−1^ (these parameters were obtained experimentally by applying to the plant a positive bounded control as explained by Zavala [14], which sets the value of D by reference to the values of 3 g COD/L and 2 g COD/L for S1, these being the maximum and minimum value that is generated from D). The concentration of organic matter in the effluent (s1) was measured on-line to estimate x1 and *Q_CH4_*. Figure 3 shows the convergence of the range observer for the concentration of anaerobic mass x1; Figure 4 shows the curves, and on-line measurement for the methane biogas flow *Q_CH4_*.

## 7. Experimental Results of the SIOS-IFD

The developed SIOS-IFD scheme is able to detect unique, sudden and permanent faults in sensors *s_1_*, *x_1_* and *Q_CH4_*, from response *r*_1_ = *y*_1_ – *ŷ*_1_ and response *r*_2_ = *y*_2_ – *ŷ*_2_, being *y_1_* = *x_1_* and *y_2_* = *Q_CH4_*.

**Figure 5.**
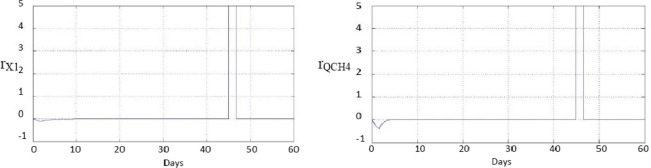
Responses to a single sudden and permanent fault, +5% in the sensor *s_1_*.

Figure 5 shows the responses to a single, sudden and permanent fault, +5% in the sensor s_1_, on day 45 of experimentation. This figure shows that both responses have faults on day 45, because both *◯*_1_ and *Q̂*_*CH*4_, have been estimated from *s_1_* so the two estimates are wrong from the moment in which the fault of *s_1_* occurs.

**Figure 6.**
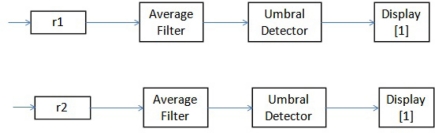
Developed response assessment scheme.

Figure 6 shows that each of the responses generated by the diagnostic scheme developed are filtered through a moving window averaging filter. Subsequently, each filteredresponse is evaluated by a threshold detector. The output of each threshold detector is connected to a display, which can only display one of the following values: −1, 0 or 1. The value “−1” indicates that the response reached a value averaged less than the threshold, so, the response had a negative average change. Similarly, the value “1” indicates that the response had an average positive change. Finally, the value “0” indicates that either the responses does not have any media change; or rather variations in their average values were below preset thresholds.

The array of symptoms presented in Figure 6 [11] is the result of the evaluation of the responses generated by the fault diagnosis scheme developed for the example of a single, sudden and permanent fault of +5% in sensor*s_1_*. These responses were shown in Figure 5.

Table 2 presents the Structured Diagnostic Matrix for unique faults in the sensors *s_1_*, *x_1_* y *Q_CH4_*, obtained from the developed response assessment scheme.

Shaded In Table 2 is symptom vector [11], which is the result of the evaluation of the response generated by the fault diagnosis scheme developed for the example of a single, sudden and permanent fault of +5% in the sensor *s_1_*. In this same Table it can be seen that there is a different symptom vector for each single, sudden and permanent fault in sensors *s_1_*, *x_1_* y *Q_CH4_*.

## 8. Conclusions

In this article experimental and simulation results of a novel system of diagnosis of faults in sensors are presented. It has been named as SIOS-IFD (Simplified Observer Interval Scheme—Instrument Fault Detection). The main advantage of the SIOS-IFD scheme in comparison with all the other schemes presented above is the fact that the measurement does not require any input, only knowledge of the range of values that such targets can achieve is required. The SIOS-IFD scheme only allows the location of a single sensor fault, for which it requires the in-line measurement in a single output.

## Figures and Tables

**Table 1.** Model Parameters.

**Parameter**	**Value and units**
*K_m1_*	5.1 gCOD/gCOD d
*K_s1_*	0.5 gCOD/L
*K_d_*	0.02 L/d
*Y_1_*	0.1 gCOD/gCOD
*Y_CH4_*	0.35 LCH_4_/gCOD
*a*	0.5 (adimensional)

**Table 2.** Structured Diagnostic Matrix.

	**Fault *s_1_* +5%**	**Fault *s_1_* −5%**	**Fault *x_1_* +5%**	**Fault *x_1_* −5%**	**Fault *Q_CH4_* +5%**	**Fault *Q_CH4_* −5%**
r_1_	1	−1	1	−1	0	0
r_2_	1	−1	0	0	1	−1
